# Rituximab treatment of adults with primary focal segmental glomerulosclerosis

**DOI:** 10.1038/s41598-023-33678-y

**Published:** 2023-04-25

**Authors:** Liuwei Wang, Lu Yu, Yulin Wang, Yanhong Guo, Zihan Zhai, Lin Tang

**Affiliations:** Department of Nephrology, Zhengzhou University First Affiliated Hospital, 1 Jianshe Road, Zhengzhou, 450052 Henan China

**Keywords:** Focal segmental glomerulosclerosis, B-1 cells

## Abstract

To evaluate the efficacy and safety of rituximab (RTX) in the treatment of primary focal segmental glomerulosclerosis (FSGS) in adults. The clinical data of patients with primary FSGS who received RTX treatment in the First Affiliated Hospital of Zhengzhou University were analyzed retrospectively. The selected patients received RTX twice or four times, with a single dose of 375 mg/m^2^, and the interval between two times of administration of RTX was 2–4 weeks. The treatment target is to achieve the clearance of B cells (peripheral blood B cell count < 5/μl). The primary outcome measures were remission and recurrence of renal disease, and the secondary outcome measures were adverse events and renal outcomes. A total of 14 FSGS patients were included, including 12 males, 9 with glucocorticoid-dependent or frequently relapsing nephrotic syndrome, and 3 with newly diagnosed nephrotic syndrome. After RTX treatment, 7 patients with glucocorticoid-dependent/recurrent nephrotic syndrome were completely relieved. At 6 months of follow-up, glucocorticoids were discontinued in all patients except 1 patient. The other 5 patients achieved partial remission (PR), of which 1 patient relapsed after PR, and 1 initial patient achieved complete remission. One patient progressed to end-stage renal disease (ESRD) after 4 months of follow-up. RTX in the treatment of adult glucocorticoid-dependent/relapsing FSGS can reduce the risk of recurrence and help to decline or discontinue the use of glucocorticoid and immunosuppressants.

## Introduction

Focal segmental glomerulosclerosis (FSGS) is a common pathological type of primary nephrotic syndrome in children and adults, which requires high-dose glucocorticoid and immunosuppressive therapy^1^. The long-term presence of nephrotic syndrome indicates poor prognosis, with 50% of patients progressing to end-stage renal disease (ESRD) within 6 to 8 years^2^.

Glucocorticoid is the first-line drug for the treatment of FSGS. About 2/3 of patients are effectively treated with glucocorticoid^1^, but more than 50% of them relapse during glucocorticoid reduction or after drug withdrawal^3^. FSGS is a characteristic histopathological change common to many glomerular diseases, and its main target lesion is podocyte. The clinical manifestation of idiopathic FSGS is nephrotic syndrome, and glucocorticoid is the cornerstone of treatment^4^^.^ Domestic studies reported that the complete remission (CR) rate of FSGS patients in early adulthood was 25.8%, and the total effective rate was 83.9%. In addition to patients with glucocorticoid resistance, some patients experienced recurrence or glucocorticoid dependence during treatment. Regardless of single glucocorticoid treatment or long-term glucocorticoid combined with immunosuppressant, patients will have greater adverse reactions. For patients with glucocorticoid resistance, relapse or glucocorticoid dependence, not only is the efficacy of simple glucocorticoid poor, but also immunosuppressants (such as cyclosporine A, tacrolimus, mycophenolate mofetil, cyclophosphamide, etc.) are usually added. Some patients cannot achieve clinical remission after the addition of immunosuppressants. The efficacy characteristics and adverse reactions of such drugs limit their long-term use, and about 20% of patients are still ineffective to the above treatment, and will progress to ESRD within 3–5 years. New approaches are urgently needed to solve the dilemma of FSGS treatment.

Rituximab (RTX) is a human-mouse chimeric monoclonal antibody, which can specifically bind to CD20 molecules on the surface of B cells^5^, eliminate B cells through antibody or complement-mediated cytotoxicity, apoptosis induction and other ways, and is safe and effective for the treatment of a variety of immune diseases. The results of some observational studies and controlled clinical trials have shown good efficacy of RTX in the treatment of minimal change nephropathy^6,7^ and nephrotic syndrome caused by primary membranous nephropathy^8,9^ .There are few clinical studies on the treatment of FSGS patients with RTX in China and abroad^10^, and most of them have limitations such as small sample size and inconsistent treatment plans^11–19^. We retrospectively analyzed the clinical data of patients with primary FSGS treated with RTX to evaluate the efficacy and safety of RTX in the treatment of FSGS.

## Methods

### Data source and study population

Adult patients with primary FSGS nephrotic syndrome admitted to the Third Department of Renal Internal Medicine of the First Affiliated Hospital of Zhengzhou University from December 2019 to August 2022 were included in this single-center, retrospective analysis study. All participants were treated with RTX and regularly followed. The clinical manifestations of refractory nephrotic syndrome include steroid-dependent nephrotic syndrome (SDNS), frequently relapsing acute nephrotic syndrome (FRNS), steroid persistent nephrotic syndrome (SRNS), or the presence of contraindications to steroid use. SDNS was defined as steroid reduction or relapse within 2 weeks after drug withdrawal. SRNS was defined as nephrotic syndrome with no remission after prednisone (1 mg·kg^−1^·d^−1^) treatment for more than 12 weeks. FRNS was defined as 2 or more relapses within 6 months.The study was approved by the Ethics Committee of the First Affiliated Hospital of Zhengzhou University and all methods were performed in accordance with the guidelines and regulations. Informed consent in writing was obtained from all study participants.

### RTX regimen

RTX was administered once or twice at a single dose of 375 mg/m^2^, with an interval of 2–4 weeks. The treatment target is to achieve the clearance of B cells (peripheral blood B cell count < 5/μl). RTX can be used repeatedly for relapse of the nephrotic syndrome and reconstitution of peripheral-blood B-cell (peripheral blood B cell count again ≥ 5/μl).

### Data collection

Demographic and clinical data of the selected persons were collected by consulting the electronic medical record system and telephone call back visit. Data of serum albumin, serum creatinine (Scr) and urinary protein/creatinine ratio (uPCR) at 0, 3, 6 months were collected, as well as peripheral blood B cell count during the follow-up.

### Outcome variables

The primary outcome measures of follow-up were the remission and recurrence of renal disease, and the secondary outcome measures included adverse events (AEs) and renal outcomes. CR was defined as uPCR < 0.3 g/g, serum albumin > 35 g/L and stable Scr. Partial remission (PR) was defined as uPCR < 3.5 g/g or a decrease of more than 50% from the peak value, while serum albumin level increased, and Scr was stable or increased by less than 30% higher from the baseline value^9^. Recurrence was defined as recurrence of uPCR ≥ 3.5 g/g after a complete or PR.

### Statistical analysis

SPSS22.0 software was used for statistical analysis of data. Continuous variable with normal distribution was described by x ± s and comparison between the two groups was performed by t test. Continuous variable with non-normal distribution was expressed as M (P25, P75). Classification variables were expressed in frequency and/or percentage. *P* < 0.05 indicated that the difference was statistically significant.

## Results

### Study population

A total of 14 adult patients with primary FSGS were enrolled in this study, including 12 males. The median age at first RTX administration was 30.5 (18, 73) years. Nine patients presented with SDNS or FRNS, and 8 of them recurred after treatment with multiple tacrolimus. The other patient was treated with RTX plus cyclosporine A (the trough concentration of cyclosporine A was 100–200 μg/L) (Table 1). Three patients achieved CR before RTX treatment, and the remaining patients presented with nephrotic level proteinuria, uPCR4.68 (3.9, 6.1) g/g. One case had elevated serum creatinine (354 μmol/L) and FSGS with acute tubular necrosis confirmed by renal biopsy. The baseline Scr was 83 (72.3,138.0) μmol/L. Renal pathology showed 8 cases of non-special type, 1 case of portal type, 5 cases of apical type, and 14 cases of glomerulosclerosis, ranging from 0.2% to 52.0%. (Tables 1 and 2).

**Table 1:** Clinical data of the 14 patients with primary FSGS.

Case serial number	Age (year)	Gender	Disease course (year)	Previous treatment	clinical features	RAAS antagonist
Example 1	66	M	6	St, TAC, CTX	SDNS	Yes
Example 2	23	M	8	St, TAC,CTX,MMF	SDNS	No
Example 3	73	M	8	St, TAC	SDNS	No
Example 4	18	M	4	St, TAC	SDNS	No
Example 5	30	M	6	St, TAC,MMF	SDNS	No
Example 6	18	M	5	St, TAC	SDNS	No
Example 7	44	F	3	St, TAC,MMF	SRNS	yes
Example 8	25	M	8	St, TAC,MMF, CsA	SDNS	yes
Example 9	45	M	7	St, TAC	SRNS	No
Example 10	31	M	20	St, TAC,MMF	FRNS	No
Example 11	18	M	1	St, TAC,CTX	first-episode	No
Example 12	30	M	1	St, TAC,CTX,MMF	first-episode	No
Example 13	64	M	1	St, TAC	first-episode	yes
Example 14	50	M	2	St, TAC	first-episode	yes

*St* Glucocorticoid, *TAC* Tacrolimus, *CsA* Cyclosporine, *MMF* Mycophenolate ester, *CTX* Cyclophosphamide, *SDNS* Steroid-dependent nephrotic syndrome, *FRNS* Frequently relapsing acute nephrotic syndrome, *SRNS* Steroid persistent nephrotic syndrome, *RAAS* Renin angiotensin aldosterone system.

**Table 2:** Treatment of 14 patients with primary FSGS.

Case serial number	Rituximab dose (mg)	Follow-up (month)	Relieve the situation
Example 1	2	5	PR
Example 2	2	12	CR
Example 3	2	6	CR
Example 4	1	5	CR
Example 5	2	11	CR
Example 6	1.5	11	CR
Example 7	2	10	PR
Example 8	2	10	CR
Example 9	1	5	ESRD
Example 10	2	7	PR
Example 11	2	2	CR
Example 12	2	13	CR
Example 13	1	6	CR
Example 14	2	3	PR

*PR* Partial remission, *CR* Complete remission, *ESRD* End-stage renal disease.

### RTX treatment

Ten patients received two cycles of RTX, with a total dose of 2000 mg. Two patients received a single dose of RTX, with a total dose of 1000 mg. The median time for 13 patients to achieve the clearance of B cells after the first administration of RTX was 17.5 (14.5, 20.0) days. B cell reconstitution occurred in 5 patients, and the median time of B cell reconstitution was 97.5 (75.0, 240.0) days.

### Characteristics and treatment response of patients given RTX

The median follow-up time was 5.0 (3.0, 13.0) months. 13 patients achieved remission, including 9 CR and 4 PR. The median time to remission after the first administration of RTX was 20 (15, 150) days. The total number of recurrences was 1, and the total number of recurrences/total follow-up month was 0.01 times/month, of which 1 case had recurrence ≥ 1 within 6 months of follow-up. 9 patients achieved CR after receiving RTX treatment. The total follow-up time was 71 months, and the total number of recurrences was 0. At 6 months of follow-up, steroids were discontinued in all but one patient. One patient developed ESRD after 4 months of follow-up. Table 3 shows changes of blood biochemical indexes during the follow-up. After RTX treatment, 5 patients had B cell reconstitution, with a median time of 97.5 (75.0, 240.0) days.

**Table 3:** Follow-up data of blood and biochemical indexes of patients after RTX treatment.

Biochemical indicator	3 months	6 months	9 months
Scr (μmol/L)			
Example 1	76	65	N/A
Example 2	42	67	72
Example 3	131	130	N/A
Example 4	80	76	N/A
Example 5	78	76	82
Example 6	89	85	81
Example 7	63	61	65
Example 8	82	76	80
Example 9	51	N/A	N/A
Example 10	58	56	N/A
Example 11	62	N/A	N/A
Example 12	57	62	65
Example 13	96	100	N/A
Example 14	88	N/A	N/A
uPCR(g/g)			
Example 1	2.16	1.75	N/A
Example 2	0.57	0.42	0.30
Example 3	2.01	1.34	N/A
Example 4	0.45	0.42	N/A
Example 5	0.10	0	0
Example 6	0.11	0.11	0.13
Example 7	2.55	3.63	1.77
Example 8	0.04	0.04	0
Example 9	0.64	N/A	N/A
Example 10	2.77	4.62	N/A
Example 11	0.17	N/A	N/A
Example 12	0.08	0.05	0.1
Example 13	1.72	0.82	N/A
Example 14	1.93	N/A	N/A
ALB (g/L)			
Example 1	30.9	33.2	N/A
Example 2	47.8	49.4	48.4
Example 3	36.6	34.2	N/A
Example 4	38.1	40.8	N/A
Example 5	51.5	50.4	48.3
Example 6	38.9	47.5	45.2
Example 7	34.1	30.1	36.4
Example 8	37.3	42.6	43.2
Example 9	24.1	N/A	N/A
Example 10	30.3	18.2	N/A
Example 11	49.3	N/A	N/A
Example 12	43.8	54.2	53.1
Example 13	32.6	38.4	N/A
Example 14	34.7	N/A	N/A

### Adverse events

One patient had a recurrence of kidney disease. No infusion reaction, serious infection or malignant tumor occurred during the follow-up.

## Discussion

This study retrospectively summarized the clinical experience of RTX in the treatment of 14 adult patients with primary FSGS. The recurrence rate of 9 SDNS/FRNS patients was magnificently reduced after the use of RTX, and the dose of glucocorticoid/immunosuppressant was withdrawn or reduced. Kronbichler et al^13^. reported that 5 adult patients with SDNS/FRNS caused by minimal change nephropathy or FSGS were treated with RTX (375 mg/m^2^) for 4 weeks. The recurrence rate of nephropathy in these patients was significantly reduced, and the complete withdrawal of immunosuppressants was significantly increased. A multi-center study in Italy showed that 30 patients with SDNS/FRNS (including 8 FSGS patients) were treated with 1 to 2 doses of RTX (375 mg/m^2^), and the total number of relapses was reduced to 25% before the treatment with RTX, and the daily glucocorticoid dosage was reduced from 0.27 mg/kg to complete withdraw^7^. It has been reported in the literature that a 4-week dose (375 mg/m^2^) of RTX was similar to a 2-week dose (375 mg/m^2^) in inducing renal remission and B cell clearance^7,12^. Considering the cost and safety factors, we adopted a 2-week dose of RTX (375 mg/m^2^)^20^.

In this study, 8 adult patients with SDNS/FRNS were relieved after using RTX, the recurrence rate was significantly reduced, and the dosage of glucocorticoid and immunosuppressant was significantly reduced, which was consistent with the above results, and further confirmed the efficacy of RTX in the treatment of adult glucocorticoid-dependent FSGS. At present, few children^17–19^ or adults^14,15^ with glucocorticoid resistant FSGS benefit from RTX treatment. A large cohort study reported that only 2/8 of glucocorticoid resistant FSGS patients showed improved renal function and decreased urinary protein level, and one patient had a short-term clinical benefit^16^. At present, some scholars believe that the poor response of SRNS patients to RTX may be related to gene mutation, T cell activation and age^11,12,21^. In addition, high levels of urinary protein can promote the excretion of RTX through urine, thus reducing the therapeutic effect. However, the above conclusions need further research and demonstration. In this study, all patients achieve the clearance of B cells after receiving 1–2 doses of RTX. The clearance of B cells in most patients lasted ≥ 6 months, which was slightly shorter than the standard dose or higher dose in the above study.

Recent evidence showed that B cell reconstitution was related to disease recurrence, but in a few patients, B cell reconstitution occurred after disease recurrence, which may be related to insufficient clearance of B cells from lymphatic organs and tissues by RTX^6^. It is suggested that the change of peripheral blood B cell count is not enough to judge the recurrence of FSGS patients. Current studies have shown that RTX is well tolerated and safer than traditional treatment schemes such as glucocorticoid in most cases^6,13,22^. However, since RTX can lead to low humoral immune function and secondary serious infection^20^, serum immunoglobulin should be regularly monitored before and after the use of RTX to timely assess the risk of infection. Moreover, some rare but serious AEs should be noted when using RTX in clinical practice, such as progressive multifocal leukoencephalopathy, myocardial infarction and fatal heart failure^23^.

A new meta-analysis in 2021 showed that a CR rate of 43% in adult FSGS treated with RTX, and a recurrence rate of 29.8%^24^. Safety analysis showed that RTX was well tolerated. In general, RTX is superior to steroid-resistant FSGS for recurrent or steroid-dependent FSGS, and to adults for children. In addition, studies have shown that RTX is effective for FSGS patients with ineffective initial treatment or plasma exchange, and the prophylactic use of RTX can reduce the incidence of post-transplant albuminuria in high-risk FSGS patients^25,26^.

RTX can effectively induce remission of glucocorticoid-dependent/recurrent primary FSGS nephrotic syndrome in adults^24^, reduce the risk of recurrence, and reduce the dosage of glucocorticoid and immunosuppressant or withdraw them. However, for patients with clinical manifestations of SRNS, especially those with immunosuppressant resistance, the effect is still poor. Further studies are needed to determine which types of SRNS patients may benefit from the treatment of RTX. For FSGS patients with clinical manifestations of SRNS, if RTX is required, the clinical benefits and risks of the treatment plan should be fully evaluated. Limitations need to be acknowledged. This study included a small number of patients, had a short follow-up time, and was limited to the retrospective nature.

## Conclusion

In conclusion, RTX appears to effectively decrease relapses, and RTX treatment can achieve CR or PR of proteinuria in patients with FSGS or MCD in a cost-effective manner. Our results are particularly remarkable given that all patients had failed prior to RTX treatment. More prospective randomized controlled trials of RTX are needed to truly assess efficacy and durability of this drug compared with traditional specific immunosuppressive therapy.

## Data Availability

The data underlying this article are available in the article.
